# An optimized method for synthesis and purification of 1,1,6-trimethyl-1,2-dihydronaphthalene (TDN)

**DOI:** 10.1016/j.mex.2019.12.009

**Published:** 2019-12-16

**Authors:** Alexey Dobrydnev, Andrii Tarasov, Nikolaus Müller, Yulian Volovenko, Doris Rauhut, Rainer Jung

**Affiliations:** a Department of Organic Chemistry, Faculty of Chemistry, Taras Shevchenko National University of Kyiv, Lva Tolstoho Str. 12, 01033 Kyiv, Ukraine; b Department of Enology, Hochschule Geisenheim University, Von-Lade-Straße 1, 65366 Geisenheim, Germany; c Silvanerweg 9, 55595 Wallhausen, Germany; d Department of Microbiology and Biochemistry, Hochschule Geisenheim University, Von-Lade-Straße 1, 65366 Geisenheim, Germany

**Keywords:** Synthesis and purification of 1,1,6-trimethyl-1,2-dihydronaphthalene (TDN), TDN, High purity, Wine, Enology

## Abstract

1,1,6-Trimethyl-1,2-dihydronaphthalene (TDN), an aroma compound present in wine, is used for sensory and physicochemical analyses. Therefore, synthesis of TDN of high purity is required for these purposes. Optimization of TDN synthesis in order to facilitate its subsequent purification was described. As a result, ≥99.5 % of TDN purity was reached.

• The possibility of using both *α*-ionone and *β*-ionone as starting substances was demonstrated
• Modifications of the use of reagents in the second and third steps of TDN synthesis were proposed

## Graphical abstract

**Figure:**
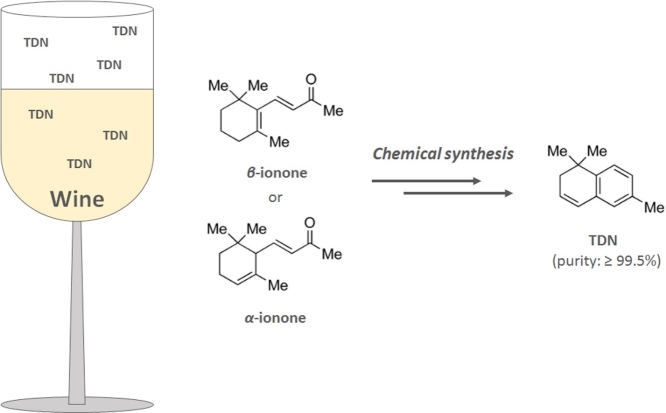

**Specification Table**

**Table:** 

Subject Area:	Chemistry
More specific subject area:	Organic Chemistry
Method name:	Synthesis and purification of 1,1,6-trimethyl-1,2-dihydronaphthalene (TDN)
Name and reference of original method:	Miginiac, P. (1990). A Facile Synthesis of 1,1,6-Trimethyl-1,2-Dihydronaphthalene. *Synth. Commun*., 20(12), 1853–1856. https://doi.org/10.1080/00397919008053110
Resource availability:	The data is available in the article

## Method details

### Background

1,1,6-Trimethyl-1,2-dihydronaphthalene (TDN) is an aroma substance which is typically found in wines [1,2]. TDN has a controversial sensory effect on the wine bouquet since its aroma is usually associated with kerosene and petrol notes. Comparing wines made of various grape varieties, a perceivable amount of TDN is found mostly in Riesling wines. Precursors of TDN are carotenoid derived compounds originating from the grapes [1,3,4]. These precursors are slowly converted to TDN in the acidic medium of wine. Kerosene/petrol aroma usually becomes perceivable after several years of wine storage. Viticultural and enological practices as well as the bottle stoppers selection and wine storage conditions can affect the level of TDN in wines [3,[5], [6], [7], [8], [9], [10]]. Investigations of TDN content in wine become a topic of high interest due to the global climate change. Elevated temperature and intense sun exposure favor the appearance of TDN in wine at the earlier stages of aging and at higher concentrations. Therefore, study of TDN as aroma component in wine and its management draws attention of scientists nowadays.

The necessity for TDN of high purity is explained by specific needs of enological research. Results of sensory analysis of TDN can be distorted by the impurities in TDN, which reveal low perception thresholds. The presence of such impurities can affect the sensory evaluation of TDN in wines, water or ethanol solutions. Another aspect of high purity TDN application is a reference sample for GC–MS analysis of wines.

The chemical synthesis of TDN was described in 1990 by Miginiac. In the current work we optimized this method according to our experience in order to facilitate preparation and purification of TDN.

### Chemicals and equipment

Reactions requiring anhydrous conditions were performed with the usual precautions for rigorous exclusion of moisture. Tetrachloromethane (CCl_4_) anhydrous, ≥99.5 % purity, was dried by distillation from phosphorus pentoxide. The other chemicals were acquired from Enamine Ltd., Sigma-Aldrich or Fluka and, when necessary, were purified according to the reported procedure [11]: 4-(2,6,6-trimethyl-2-cyclohexenyl)-3-buten-2-one (*α*-ionone) natural, ≥86 %; 4-(2,6,6-trimethyl-1-cyclohexenyl)-3-buten-2-one (*β*-ionone) natural, ≥95 %; *N*,*N*-diisopropylethylamine (Hünig’s base) purified by redistillation, 99.5 %; cyclohexane anhydrous, 99.5 %; acetonitrile (MeCN) for HPLC, gradient grade, ≥99.9 %; water (H_2_O), HPLC grade; benzoyl peroxide (Bz_2_O_2_), reagent grade, ≥98 %; iodine (I_2_), ≥99.8 %; *N*-bromosuccinimide (NBS) for synthesis, ≥95 %; sodium disulfite (Na_2_S_2_O_5_) puriss. p.a., 98–100.5 %; sodium (Na), 99 %; sodium sulfate (Na_2_SO_4_) anhydrous, granular, ≥99 %; hydrochloric acid (aq. HCl), 37 %.

^1^H NMR spectra were recorded on a Bruker Avance 500 spectrometer at 499.9 MHz using CDCl_3_ as a solvent and Me_4_Si as an internal standard. Mass spectra were recorded with Agilent 7890 Series using electron impact ionization at 1176 V. The HPLC purification was performed on an Agilent Infinity 1260 instrument using Waters SunFire c18 190 × 10 mm 5 mcm column, Diode Array Detector VL (G1315D), and gradient elution with acetonitrile-water (from 70 % up to 100 % of acetonitrile).

### Procedure

**Fig. 1:**
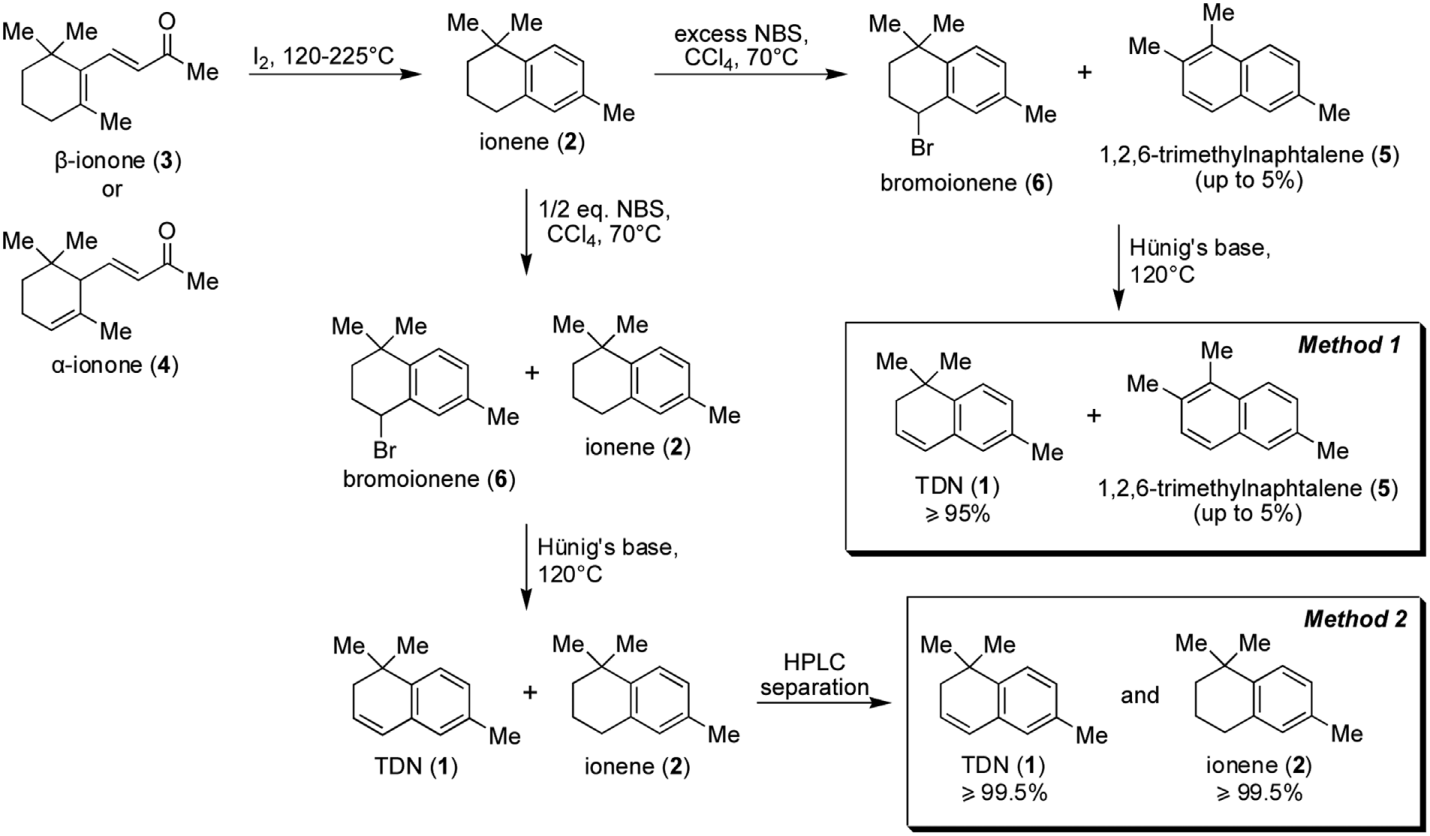
Scheme of TDN (**1**) synthesis.

Synthesis of TDN (**1**) was performed in three steps *via* formation of ionene (**2**) and bromoionene (**6**) according to the scheme illustrated in Fig. 1. Types of by-products and their amount in the final TDN (**1**) depended on the amount of NBS added in the second step of synthesis.

#### Synthesis of 1,1,6-trimethyl-1,2,3,4-tetrahydronaphthalene (ionene (**2**)), purity ≥95 %

*α*-Ionone (**4**) or *β*-ionone (**3**) (15.4 g, 80 mmol, avoid usage of larger quantities!) and iodine (0.3 g, 1.18 mmol) were placed in a 250-mL flask fitted with a thermometer dipping into the liquid. Iodine was dissolved upon stirring and mild heating. Then, the mixture was slowly heated in an oil bath until the exothermic reaction started at about 100−120 °C. At this moment the flask was removed from the oil bath. After the reaction subsided, the heating was started again and kept till the inside temperature reached 225 °C. During the heating, the water formed was distilled off until its development ceased. After cooling, the liquid remaining in the flask was dissolved in cyclohexane (75 mL) and washed with aqueous Na_2_S_2_O_5_ (1 M) (3 × 25 mL) and water (50 mL). After drying (Na_2_SO_4_) and evaporation of volatiles, the residue was distilled over sodium (0.5 g) affording 8.2 g (47 mmol, 52 % yield) of 98 % pure ionene (**2**): b.p. = 112–115 °C (15 Torr) [for spectra and physical data see ref. [12]. lit. b.p. = 125–126 °C (22 Torr)].

#### Synthesis of 1,1,6-trimethyl-1,2-dihydronaphthalene (TDN (**1**)), purity ≥95 % (**Method 1**)

Ionene (**2**) (9.11 g, 50 mmol) was dissolved in distilled CCl_4_ (100 mL) in a two-necked flask equipped with a thermometer and a reflux condenser. After addition of *N*-bromosuccinimide (11.50 g, 64.6 mmol) and benzoyl peroxide (100 mg), the mixture was heated and irradiated with a 100 W tungsten lightbulb. An exothermic reaction started at about 70 °C. Heating was stopped and as soon as the reaction subsided, it was refluxed again for 15 min. After cooling and filtration, the collected succinimide was washed with CCl_4_ (10 mL). The combined filtrates were evaporated at reduced pressure and the brown residue was distilled in vacuum without fractionation (b.p. = 50–75 °C at 0.3 Torr). Then the distillate consisting of bromoionene (**6**) and raw TDN (**1**) was placed into a 100-mL flask equipped with a reflux condenser and Hünig’s base (*N*,*N*-diisopropylethylamine) (9.68 g, 13.05 mL, 75 mmol) was added. The flask was submerged into the oil bath, gradually heated up to 120 °C and kept at this temperature for 15 min. After cooling, cyclohexane (75 mL) was added, the mixture was filtered and the organic phase was successively washed with aqueous HCl (3 M) (3 × 25 mL) and H_2_O (3 × 50 mL). After drying (Na_2_SO_4_) and evaporation of volatiles the residue was distilled over sodium (0.5 g) affording 5.0 g (28 mmol, 56 % yield) of colorless TDN (**1**) of 96 % purity (**1**). b.p. = 113–114 °C (15 Torr) [for spectra and physical data see ref. [12]. lit. b.p. = 115 °C (18 Torr)].

#### Synthesis of 1,1,6-trimethyl-1,2-dihydronaphthalene (TDN (**1**)), purity ≥99.5 % (**Method 2**)

Ionene (**2**) (18.21 g, 0.10 mol) was dissolved in distilled CCl_4_ (100 mL) in a two-necked flask equipped with a thermometer, and a reflux condenser. After addition of *N*-bromosuccinimide (11.5 g, 64.6 mmol) and benzoyl peroxide (100 mg, 0.4 mmol), the mixture was heated and irradiated with a 100 W lightbulb. An exothermic reaction started at about 70 °C. Heating was stopped and as soon as the reaction subsided, it was refluxed again for 15 min. After cooling and filtration the collected succinimide was washed with CCl_4_ (100 mL). Then the combined filtrates were evaporated at reduced pressure and placed into a 250 mL flask equipped with a reflux condenser. Hünig’s base (*N*,*N*-diisopropylethylamine) (12.9 g, 17.4 mL, 0.1 mol) was added and the resulting mixture was submerged into the oil bath, gradually heated up to 120 °C and kept at this temperature for 15 min. After cooling, cyclohexane (100 mL) was added, the mixture was filtered and the organic phase was successively washed with aqueous HCl (3 M) (3 × 25 mL) and H_2_O (3 × 50 mL). After drying (Na_2_SO_4_) and evaporation of volatiles the residue was distilled over sodium (0.5 g) affording 13.6 g of approx. 1:1 mixture of ionene (**2**) and TDN (**1**). b.p. = 113–114 °C (15 Torr).

The mixture was separated by HPLC. TDN (**1**) (GC–MS (EI): *m/z* = 172 [M]^+^) and ionene (**2**) (GC–MS (EI): *m/z* = 174 [M]^+^) were found in the first and the second fractions, respectively. The target compounds were isolated according to the following procedure. The collected fraction was evaporated at reduced pressure (15–20 Torr) up to 1/10 of the initial volume at a temperature not higher than 50 °C. The remainder was diluted with water (50 mL) and extracted with hexane (5 × 30 mL). The organic phase was dried (Na_2_SO_4_), evaporated at reduced pressure and the residue was distilled over sodium (0.2 g, 8.7 mmol) at 15 Torr affording ≥99.5 % purity specimens of TDN (**1**) (3.9 g, 22.6 mmol) and ionene (**2**) (3.7 g, 21.2 mmol).

The purity of compounds was controlled by GC–MS spectra recorded on Agilent 7890 with electron impact ionization at 1176 V.

### Results and discussion

The synthesis of TDN (**1**) was based on the protocol reported by Miginiac [12] and modified according to our experience in order to obtain analytical specimens of TDN (**1**) of high purity. No significant changes were applied to the first step: synthesis of ionene (**2**). The methodology of synthesis closely followed the protocol, except the usage of both, *β*-ionone (**3**) or *α*-ionone (**4**) as starting material (Fig. 1). Thus, these two compounds are interchangeable for the given synthesis and allowed to obtain 98 % purity specimen of ionene (**2**).

The next step was focused on the optimization of ionene (**2**) conversion to TDN (**1**). High attention was devoted to this process due to the formation of the by-product 1,2,6-trimethylnaphthalene (**5**). Being impurity with similar physical properties to TDN, 1,2,6-trimethylnaphthalene (**5**) was difficult to remove from the obtained TDN (**1**). Following the original protocol of Miginiac [12], 1,2,6-trimethylnaphthalene (**5**) was formed in the presence of excess of NBS [13] and remained in the final TDN (**1**) in a quantity up to 5 % (for its spectral data refer Mayer and Duswalt [14]). As a result, 96 % was the highest purity of TDN specimen reached by ***Method 1***.

In order to avoid side reactions caused by an excess of NBS, only half of the stoichiometric amount of NBS was added in ***Method 2*** compared to the original protocol [12]. Thereby, the resulting mixture of unreacted ionene (**2**) and bromoionene (**6**) was treated with Hünig's base affording approximately an equimolar mixture of ionene (**2**) and TDN (**1**). A final application of HPLC separation resulted in ≥99.5 % purity specimens of ionene (**2**) and TDN (**1**).

Another noteworthy modification used in both ***Methods 1 and 2*** implied utilization of *N*,*N*-diisopropylethylamine (Hünig's base) instead of *N*,*N*-diethylaniline for an HBr elimination reaction. This reagent replacement was aimed to avoid additional aromatic admixtures in the target TDN (**1**), which would complicate TDN purification.

The mentioned synthesis modifications are especially important for HPLC purification of TDN since they avoid side reactions, facilitate separation process, reduce the number of operations with the chromatographic column and minimize the consumption of eluent.

### Conclusions

The optimization of the original method of TDN preparation [12] was described. The modifications imply using reduced amount of *N*-bromosuccinimide and *N*,*N*-diisopropylethylamine instead of *N*,*N*-diethylaniline. A subsequent separation of the final mixture by HPLC allowed to obtain TDN of high purity (≥99.5 %). The proposed modifications of TDN synthesis minimize the presence of undesirable impurities in the final mixture before HPLC process and thereby facilitate TDN purification. Also it was demonstrated, that both *α*-ionone and *β*-ionone can be used as starting material.
