# Supplementary material for: An optimized method for synthesis and purification of 1,1,6-trimethyl-1,2-dihydronaphthalene (TDN)
Source: MethodsX. 2019 Dec 16;7:56–61. doi: 10.1016/j.mex.2019.12.009 (PMC6938800; doi:10.1016/j.mex.2019.12.009)
Supplement: Supplementary file 1 [file mmc1.pdf]

Supplementary Materials

An optimized method for synthesis and purification of 1,1,6-trimethyl-1,2-dihydronaphthalene (TDN)

Alexey Dobrydnev[a], Andrii Tarasov*[b], Nikolaus Müller[c], Yulian Volovenko[a], Doris Rauhut[d], Rainer Jung[b]

[a] Department of Organic Chemistry, Faculty of Chemistry Taras Shevchenko National University of Kyiv
Lva Tolstoho str. 12, 01033 Kyiv, Ukraine

[b] Department of Enology Hochschule Geisenheim University
Von-Lade-Straße 1, 65366 Geisenheim, Germany
[c] Silvanerweg 9, 55595 Wallhausen, Germany

[d] Department of Microbiology and Biochemistry Hochschule Geisenheim University
Von-Lade-Straße 1, 65366 Geisenheim, Germany

*Corresponding author's e-mail: andrii.tarasov@hs-gm.de

Table of Contents

^{1}H NMR and GC-MS Spectra Images	S2-S8
^{1}H NMR spectrum of TDN (**1**) obtained by *Method 1*, ≥ 95% purity.	S2
^{1}H NMR spectrum of TDN (**1**) obtained by *Method 2*, ≥ 99.5% purity.	S3
^{1}H NMR spectrum of Ionene (**2**) obtained by *Method 2*, ≥ 99.5% purity.	S4
GC-MS spectrum of TDN (**1**) obtained by *Method 2*, ≥ 99.5% purity	S5-S6
GC-MS spectrum of Ionene (**2**) obtained by *Method 2*, ≥ 99.5% purity	S7-S8

^{1}H NMR spectrum of TDN (1) obtained by *Method 1*, ≥ 95% purity.

The signals of 1,2,6-trimethylnaphthalene (**5**) are depicted in a selected area.

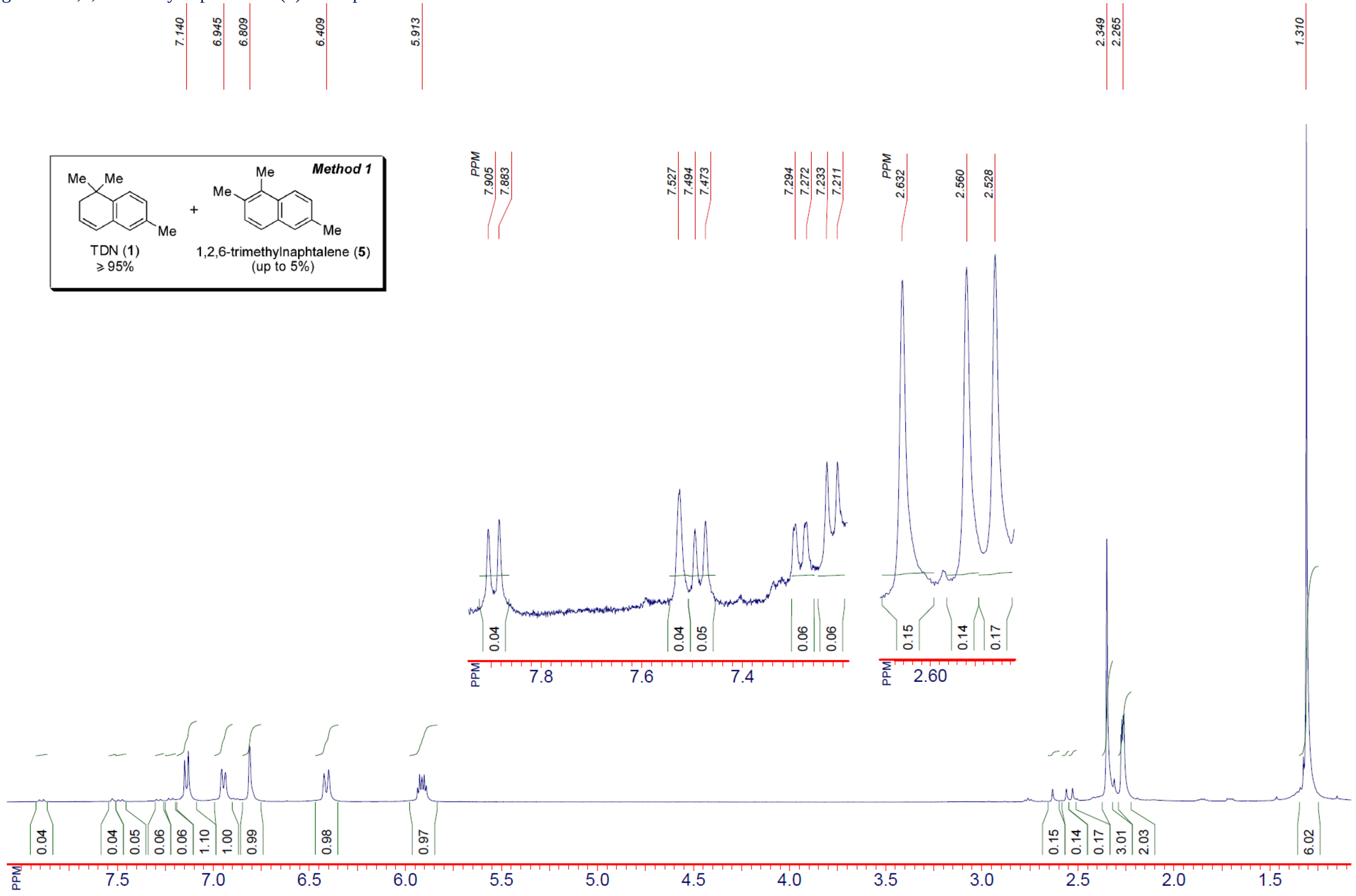

^{1}H NMR spectrum of TDN (1) obtained by *Method 2*, ≥ 99.5% purity.

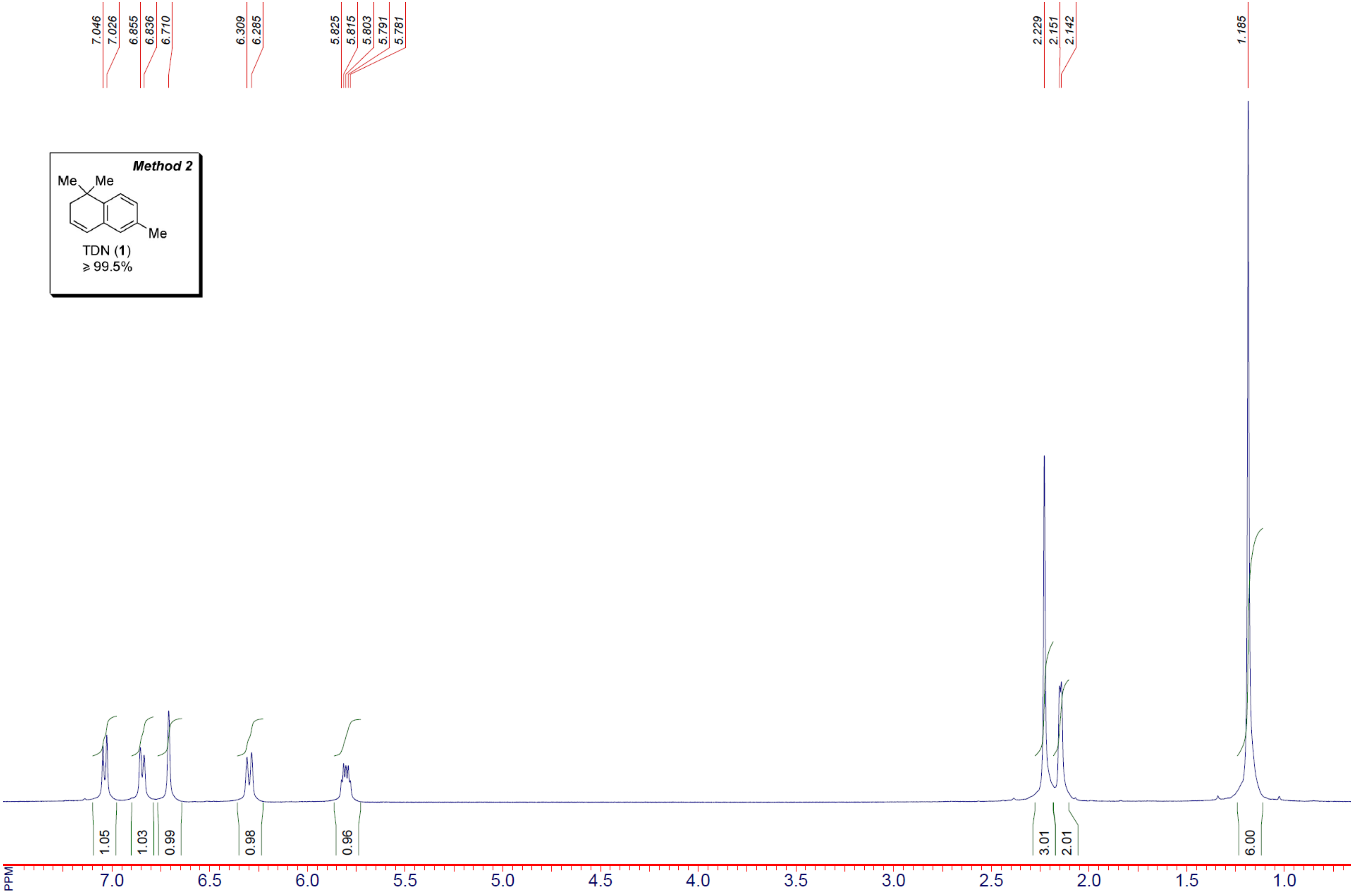

^{1}H NMR spectrum of Ionene (2) obtained by *Method 2*, ≥ 99.5% purity.

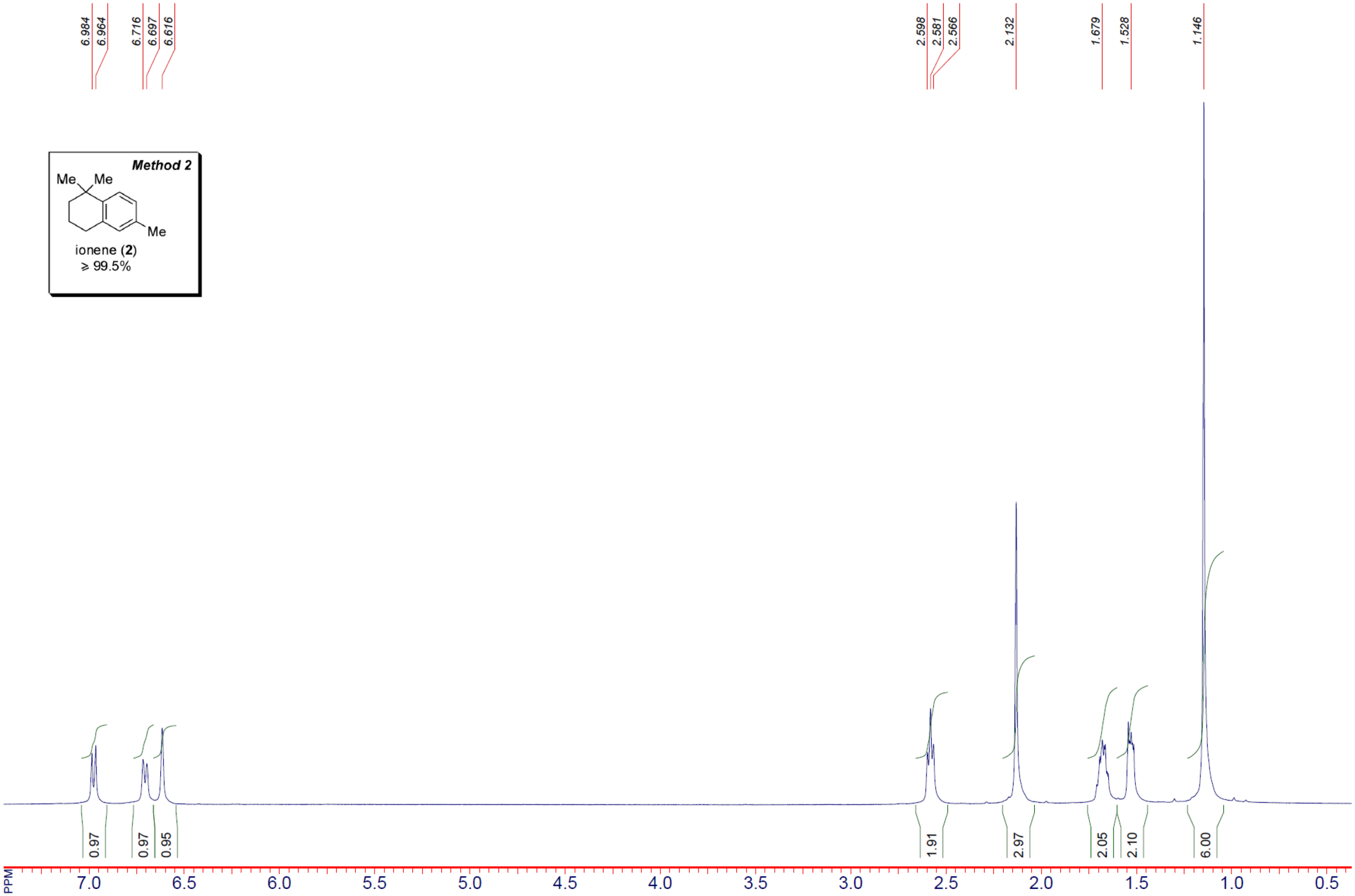

GC-MS spectrum of TDN (1) obtained by *Method 2*, ≥ 99.5% purity.

```
                                                 Library Search Report

  Data File : C:\MSDCHEM\1\DATA\05_23\UNV01559.D               Vial: 21
  Acq On    : 24 May 2017  00:19                           Operator:
  Sample    : UNV01559                                     Inst     : Instrumen
  Misc      : CH3OH                                        Multiplr: 1.00
                                                      Sample Amount: 0.00
  MS Integration Params: autoint1.e
  Method    : C:\MSDCHEM\1\METHODS\GAS_SAVE.M (Chemstation Integrator)
  Title     :
```

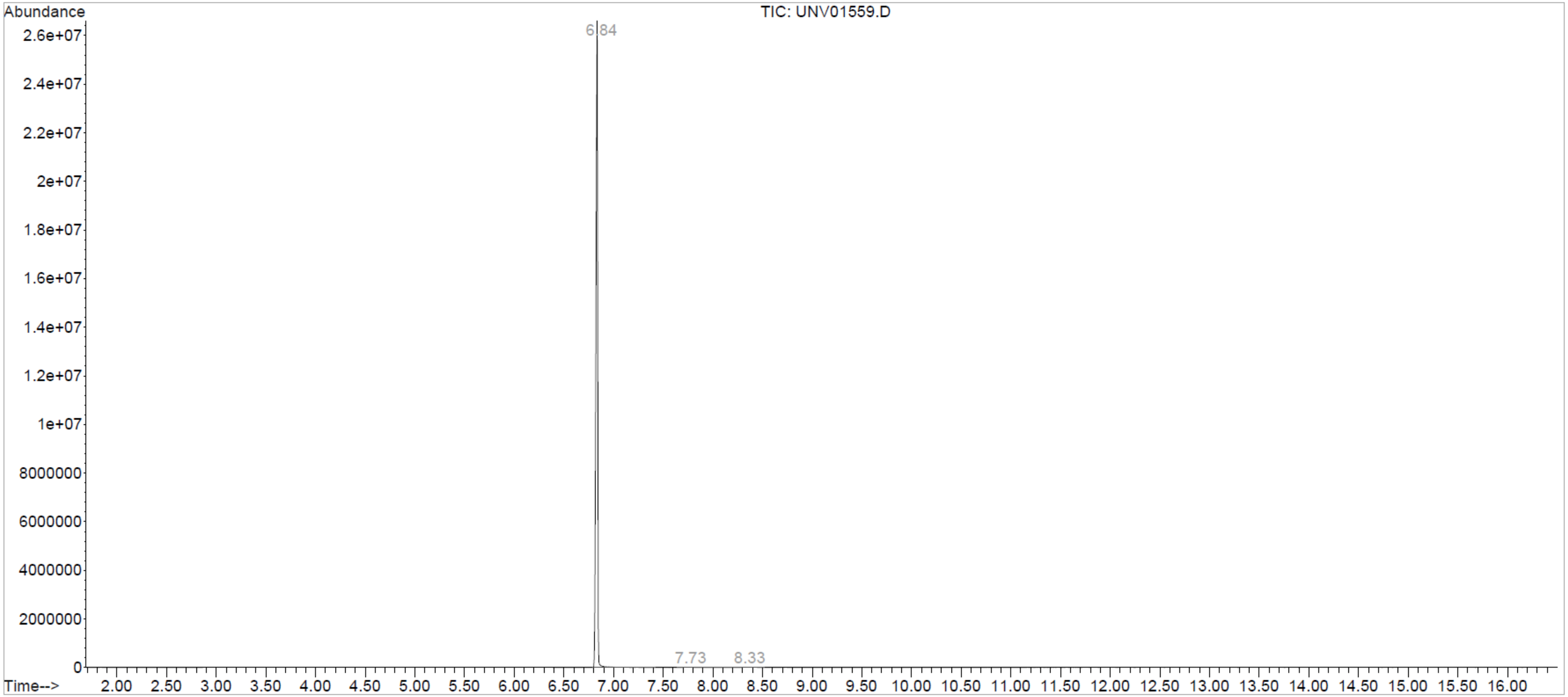

Library Search Report - Chemstation Integrator

Unknown Spectrum based on Apex

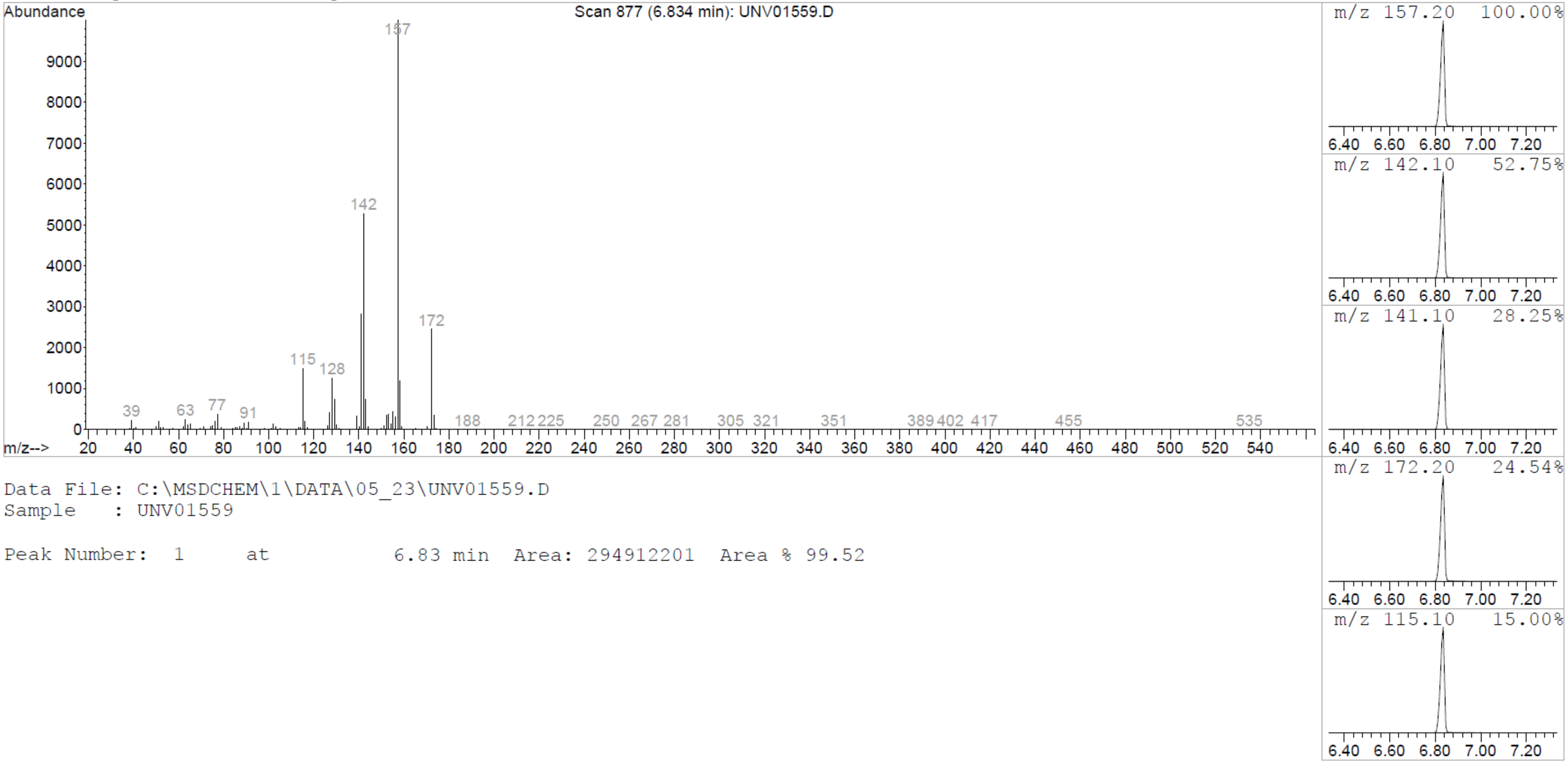

Data File: C:\MSDCHEM\1\DATA\05_23\UNV01559.D
Sample : UNV01559

Peak Number: 1 at 6.83 min Area: 294912201 Area % 99.52

GC-MS spectrum of Ionene (2) obtained by *Method 2*, ≥ 99.5% purity.

Library Search Report

```
Data Path : C:\msdchem\1\data\05 17\
Data File : UNV01557.D
Acq On    : 17 May 2017   18:38
Operator  :
Sample    : UNV01557
Misc      : CH3OH
ALS Vial  : 4   Sample Multiplier: 1

Search Libraries:   C:\Database\EMPTY.L              Minimum Quality:   0

Unknown Spectrum:   Apex
Integration Events: ChemStation Integrator - autoint1.e
```

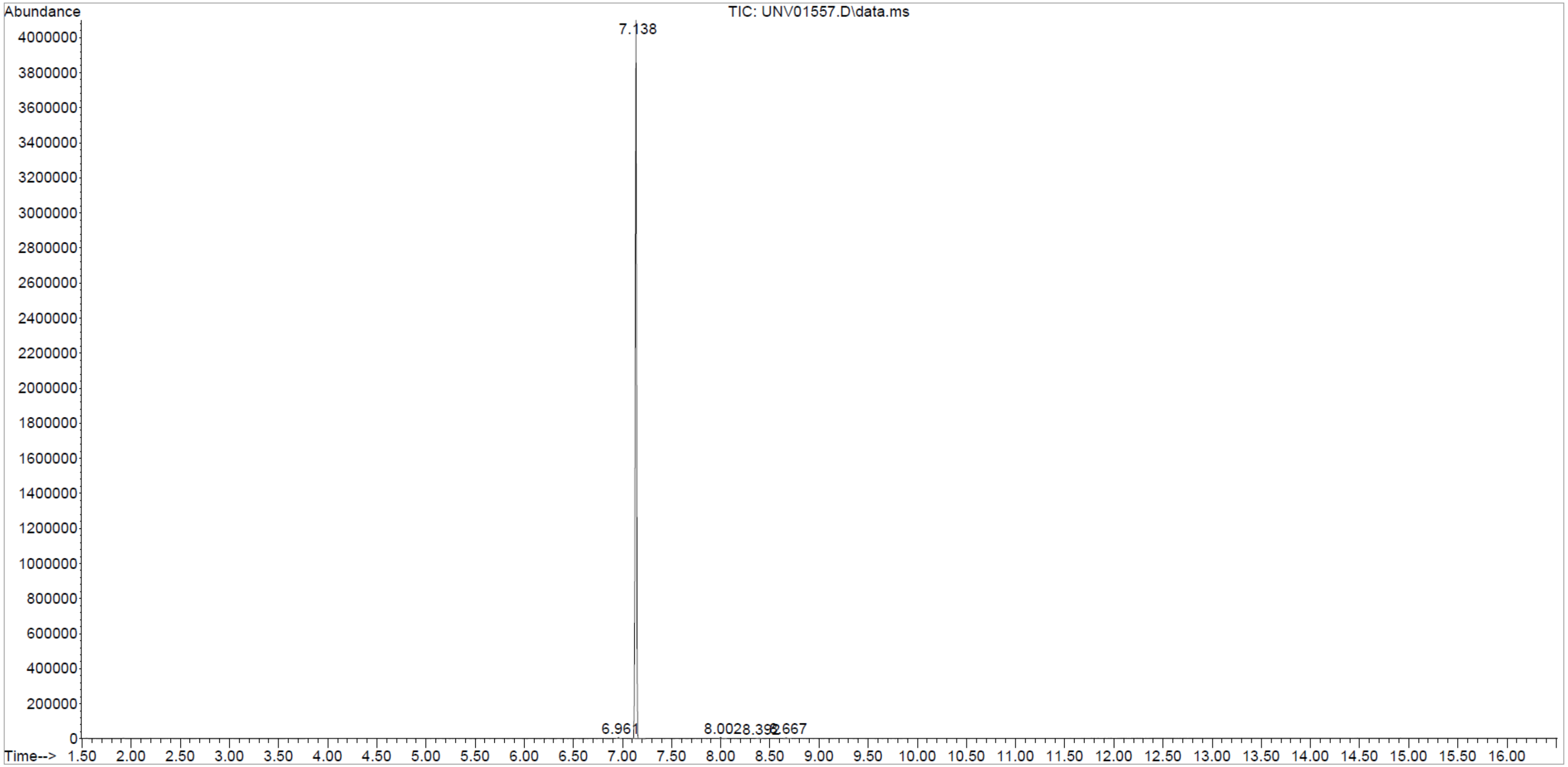

Library Search Report - ChemStation Integrator

Unknown Spectrum based on Apex

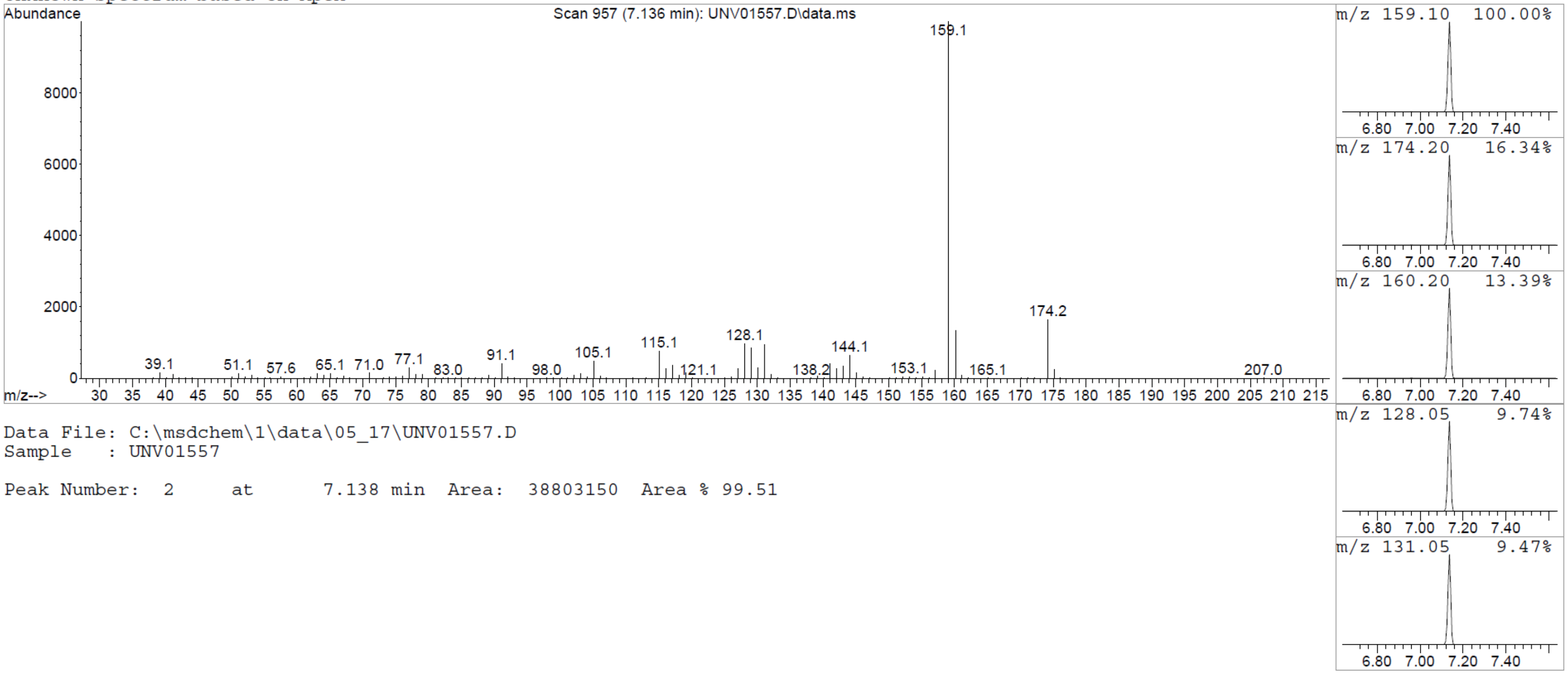

Data File: C:\msdchem\1\data\05_17\UNV01557.D
Sample : UNV01557

Peak Number: 2 at 7.138 min Area: 38803150 Area % 99.51
